# Metabolomics enables precision medicine: “A White Paper, Community Perspective”

**DOI:** 10.1007/s11306-016-1094-6

**Published:** 2016-09-02

**Authors:** Richard D. Beger, Warwick Dunn, Michael A. Schmidt, Steven S. Gross, Jennifer A. Kirwan, Marta Cascante, Lorraine Brennan, David S. Wishart, Matej Oresic, Thomas Hankemeier, David I. Broadhurst, Andrew N. Lane, Karsten Suhre, Gabi Kastenmüller, Susan J. Sumner, Ines Thiele, Oliver Fiehn, Rima Kaddurah-Daouk, for “Precision Medicine and Pharmacometabolomics Task Group”-Metabolomics Society Initiative

**Affiliations:** 1 Division of Systems Biology, National Center for Toxicological Research, U.S. Food and Drug Administration, Jefferson, AR 72079 USA; 2 School of Biosciences, Phenome Centre Birmingham and Institute of Metabolism and Systems Research (IMSR), University of Birmingham, Edgbaston, Birmingham, B15 2TT UK; 3 Advanced Pattern Analysis and Countermeasures Group, Research Innovation Center, Colorado State University, Fort Collins, CO 80521 USA; 4 Department of Pharmacology, Weill Cornell Medical College, New York, NY 10021 USA; 5 School of Biosciences, University of Birmingham, Edgbaston, Birmingham, B15 2TT UK; 6 Department of Biochemistry and Molecular Biomedicine, Faculty of Biology, Universitat de Barcelona, Av Diagonal 643, 08028 Barcelona, Spain; 7 Institute of Biomedicine of Universitat de Barcelona (IBUB) and CSIC-Associated Unit, Barcelona, Spain; 8 UCD Institute of Food and Health, UCD, Belfield, Dublin Ireland; 9 Departments of Computing Science and Biological Sciences, University of Alberta, Edmonton, AB Canada; 10 Turku Centre for Biotechnology, University of Turku, Turku, Finland; 11 Division of Analytical Biosciences and Cluster Systems Pharmacology, Leiden Academic Centre for Drug Research, Leiden University & Netherlands Metabolomics Centre, Leiden, The Netherlands; 12 School of Science, Edith Cowan University, Perth, Australia; 13 Center for Environmental Systems Biochemistry, Department Toxicology and Cancer Biology, Markey Cancer Center, Lexington, KY USA; 14 Department of Physiology and Biophysics, Weill Cornell Medical College in Qatar, Doha, Qatar; 15 Institute of Bioinformatics and Systems Biology, Helmholtz Center Munich, Oberschleißheim, Germany; 16 Discovery Sciences, RTI International, Research Triangle Park, Durham, NC USA; 17 University of Luxembourg, Luxembourg Centre for Systems Biomedicine, Campus Belval, Esch-Sur-Alzette, Luxembourg; 18 West Coast Metabolomics Center, UC Davis, Davis, CA USA; 19 Biochemistry Department, King Abdulaziz University, Jeddah, Saudi Arabia; 20 Psychiatry and Behavioral Sciences, Duke Internal Medicine and Duke Institute for Brain Sciences and Center for Applied Genomics and Precision Medicine, Duke University Medical Center, Box 3903, Durham, NC 27710 USA

**Keywords:** Metabolomics, Metabonomics, Pharmacometabolomics, Pharmacometabonomics, Precision medicine, Personalized medicine

## Abstract

**Introduction: Background to metabolomics:**

Metabolomics is the comprehensive study of the metabolome, the repertoire of biochemicals (or small molecules) present in cells, tissues, and body fluids. The study of metabolism at the global or “-omics” level is a rapidly growing field that has the potential to have a profound impact upon medical practice. At the center of metabolomics, is the concept that a person’s metabolic state provides a close representation of that individual’s overall health status. This metabolic state reflects what has been encoded by the genome, and modified by diet, environmental factors, and the gut microbiome. The metabolic profile provides a quantifiable readout of biochemical state from normal physiology to diverse pathophysiologies in a manner that is often not obvious from gene expression analyses. Today, clinicians capture only a very small part of the information contained in the metabolome, as they routinely measure only a narrow set of blood chemistry analytes to assess health and disease states. Examples include measuring glucose to monitor diabetes, measuring cholesterol and high density lipoprotein/low density lipoprotein ratio to assess cardiovascular health, BUN and creatinine for renal disorders, and measuring a panel of metabolites to diagnose potential inborn errors of metabolism in neonates.

**Objectives of White Paper—expected treatment outcomes and metabolomics enabling tool for precision medicine:**

We anticipate that the narrow range of chemical analyses in current use by the medical community today will be replaced in the future by analyses that reveal a far more comprehensive metabolic signature. This signature is expected to describe global biochemical aberrations that reflect patterns of variance in states of wellness, more accurately describe specific diseases and their progression, and greatly aid in differential diagnosis. Such future metabolic signatures will: (1) provide predictive, prognostic, diagnostic, and surrogate markers of diverse disease states; (2) inform on underlying molecular mechanisms of diseases; (3) allow for sub-classification of diseases, and stratification of patients based on metabolic pathways impacted; (4) reveal biomarkers for drug response phenotypes, providing an effective means to predict variation in a subject’s response to treatment (pharmacometabolomics); (5) define a metabotype for each specific genotype, offering a functional read-out for genetic variants: (6) provide a means to monitor response and recurrence of diseases, such as cancers: (7) describe the molecular landscape in human performance applications and extreme environments. Importantly, sophisticated metabolomic analytical platforms and informatics tools have recently been developed that make it possible to measure thousands of metabolites in blood, other body fluids, and tissues. Such tools also enable more robust analysis of response to treatment. New insights have been gained about mechanisms of diseases, including neuropsychiatric disorders, cardiovascular disease, cancers, diabetes and a range of pathologies. A series of ground breaking studies supported by National Institute of Health (NIH) through the Pharmacometabolomics Research Network and its partnership with the Pharmacogenomics Research Network illustrate how a patient’s metabotype at baseline, prior to treatment, during treatment, and post-treatment, can inform about treatment outcomes and variations in responsiveness to drugs (e.g., statins, antidepressants, antihypertensives and antiplatelet therapies). These studies along with several others also exemplify how metabolomics data can complement and inform genetic data in defining ethnic, sex, and gender basis for variation in responses to treatment, which illustrates how pharmacometabolomics and pharmacogenomics are complementary and powerful tools for precision medicine.

**Conclusions: Key scientific concepts and recommendations for precision medicine:**

Our metabolomics community believes that inclusion of metabolomics data in precision medicine initiatives is timely and will provide an extremely valuable layer of data that compliments and informs other data obtained by these important initiatives. Our Metabolomics Society, through its “Precision Medicine and Pharmacometabolomics Task Group”, with input from our metabolomics community at large, has developed this White Paper where we discuss the value and approaches for including metabolomics data in large precision medicine initiatives. This White Paper offers recommendations for the selection of state of-the-art metabolomics platforms and approaches that offer the widest biochemical coverage, considers critical sample collection and preservation, as well as standardization of measurements, among other important topics. We anticipate that our metabolomics community will have representation in large precision medicine initiatives to provide input with regard to sample acquisition/preservation, selection of optimal omics technologies, and key issues regarding data collection, interpretation, and dissemination. We strongly recommend the collection and biobanking of samples for precision medicine initiatives that will take into consideration needs for large-scale metabolic phenotyping studies.

## Introduction

In 2016, the White House announced the precision medicine initiative (PMI) in the USA to help enable a new era of individualized care through cooperative efforts by researchers, providers, and patients. The National Institutes of Health and its director Francis Collins have called for communities of researchers from around the country to make the case as to what set of technologies and disciplines would afford the highest level of efficacy in the development of the PMI. An enabling structure for this initiative has been created and partially funded (https://www.nih.gov/precision-medicine-initiative-cohort-program; Collins and Varmus 2015). Metabolomics offers a powerful set of tools, strategies, and methods necessary for the generation of complex and robust data sets, which complement data obtained by genomics and other omics methodologies and that uniquely captures effects of environment, exposome, gut microbiome and genome on human health. The Metabolomics Society’s Precision Medicine and Pharmacometabolomics Task Group herein provides a rationale as to why metabolomics is a vital and necessary component of the PMI, and for all global precision medicine initiatives going forward.

**Fig. 1:**
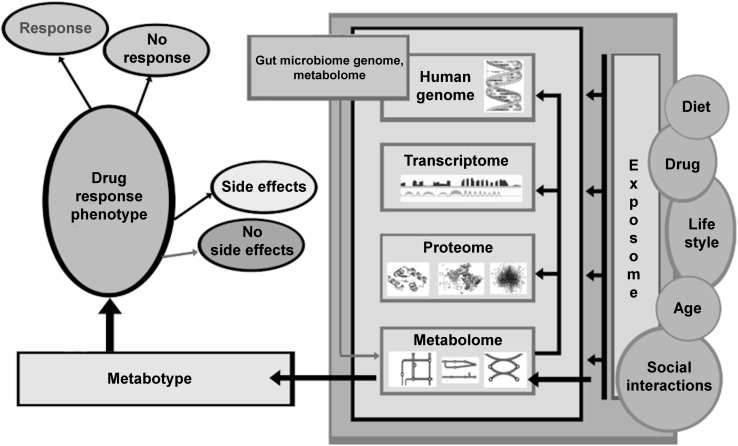
Metabolomics, a global biochemical approach for disease sub classification and drug response phenotyping

The phenotypic outcome of the complex interactions between genotype, lifestyle, diet, nutrition, drug therapy, environmental exposure, and gut microflora can now be investigated at the molecular level by identifying and quantifying a broad range of endogenous and exogenous metabolites (Fig. 1). Such metabolic phenotyping studies are able to provide new insights into disease pathophysiology, and mechanisms that underlie differences in drug responses in the human population, which contribute to predicting both risk of toxicity and beneficial responses to drug treatment (Beger et al. 2015; Cacciatore and Loda 2015; Everett 2015; Kaddurah-Daouk et al. 2008; Kaddurah-Daouk and Weinshilboum 2014, 2015; Kastenmüller et al. 2015; Nicholson et al. 2012; Patti et al. 2012; Su et al. 2014; Suhre et al. 2016; Wilson 2009; Zamboni et al. 2015). Below we provide background information, concepts related to the use of metabolism data for disease and patient sub-classification, technologies that are available for metabolic profiling, their strengths, limitations and bottlenecks, and presently available tools for large scale studies in precision medicine. We highlight how the metabolome complements and reveals details about the downstream effects of the genome, how it can describe the real-time activity of the gut microbiome, the broad effects of environmental influences that impact human health, and individual responses to treatment.

## Background

### Metabolomics and the Central Dogma

Recognition of the *Central Dogma* of molecular biology had a major impact in the life sciences. Indeed, an appreciation for the fact that life arises from chromosomal DNA being transcribed into RNA, which is in turn translated into functional proteins, affords a molecular explanation for the basis of life. It also provided molecular insights into the inherent variation in human susceptibility to diseases and for the differential efficacy of therapeutic drugs in patients. However, the *Central Dogma* fails to consider that the actual function of genes, transcripts, and proteins is specifically to control the small molecule composition of cells. It is these small molecules that carry out the main work of functioning cells, including regulating the activity of the macromolecules in a complex feedback circuit (Fig. 1). Indeed, it is the interactions of the small molecules with the macromolecular components of the cell that comprise the main determinants of cell function and dysfunction.

Importantly, the metabolome is dynamic and rich, arising only partially as the product of our own gene-encoded proteins. It also arises from the metabolic products of the microbes within us, the air we breathe, the food we eat, and the water we drink (Nicholson et al. 2004; Goodacre 2007; Scalbert et al. 2009; Lindon and Nicholson 2014). The metabolome is thus, incompletely defined and cannot be predicted from knowledge of the human genome, transcriptome, or proteome alone. Yet, despite this complexity and its associated technical challenges to comprehensive quantification, we posit that the metabolome offers the most revealing real time insights toward understanding human disease heterogeneity and variation in response to treatment, and does so at a systems level. It captures important influences on human health that go beyond the genome.

Recent technological breakthroughs have now enabled broad and confident quantification of hundreds of metabolites or relative quantification of thousands of distinct metabolites in complex biological mixtures (e.g., blood and urine). This capability advances a powerful new analytical reality of untargeted metabolite profiling. With the continuing scale-up of current technologies, bolstered by further technological advances on the near horizon, we posit that comprehensive metabolite profiling, applied to samples from large initiatives like the NIH precision medicine initiative cohort (PMI), will provide perhaps one of the most valuable tools for patient stratification, further enabling the promise of precision medicine. Accordingly, to obtain optimal return on investment in large precision medicine initiatives, it is critical that metabolomic analyses be included. While the inclusion of metabolomics could be staged over time, we urge that samples for current studies be collected in a manner that meets metabolomic profiling needs (sample collection, preparation, and storage).

### Metabotyping is currently used to screen for inborn errors of metabolism

Metabolism data and inclusion of a targeted metabolic profile in blood has led to the identification of many inborn errors of metabolism (IEMs), a group of monogenetic metabolic disorders that can be lethal in newborns, or result in irreversible organ damage, if not diagnosed and treated swiftly. However, if IEMs are recognized by early screening, many can be controlled with life-saving nutritional supplements and dietary interventions. Using current metabolite profiling platforms, which can now survey thousands of metabolites in microliter quantities of neonatal blood, we anticipate an enormously expanded scope of IEM diagnosis and the discovery of previously unrecognized genetic diseases in the near-term (Schulze et al. 2003; Vernon 2015; Yoon 2015).

### Metabotypes for genotypes—the metabolome provides a readout for functions of genetic variants

Over 100 years ago, Archibald Garrod conjectured that “inborn errors of metabolism” are “merely extreme examples of variations of chemical behavior which are probably everywhere present in minor degrees” and that this “chemical individuality predisposition to and immunities from the various mishaps which are spoken of as diseases”. Population based studies collected demographic, health and life‐style related information from thousands of individuals from the general population, and bio‐banked samples of blood, urine, and other body fluids are now analyzed using genomics, transcriptomics, proteomics, metabolomics and other large scale omics technologies (Kastenmüller et al. 2015; Sanseau et al. 2012; Shin et al. 2014; Suhre et al. 2011a, b, 2016; Suhre and Gieger 2012).

**Fig. 2:**
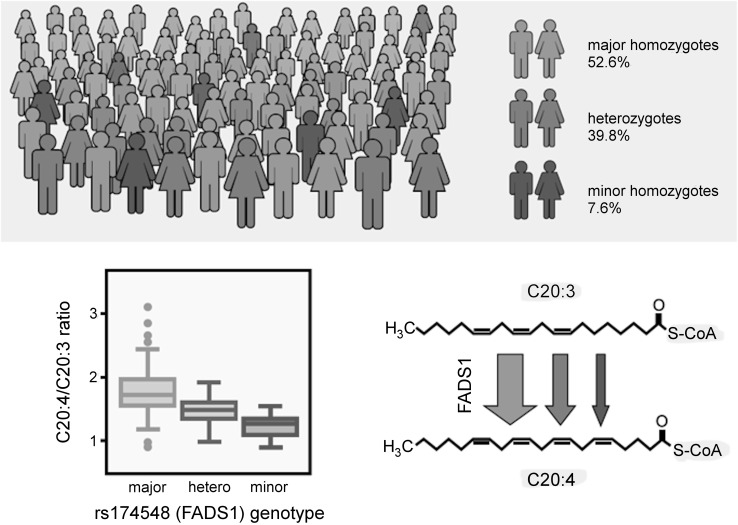
Example of a genetically influenced metabotype (GIM). Fatty acid desaturase 1 (FADS1) catalyzes the desaturation of dihomolinolenoyl-CoA to arachidonoyl-CoA (C20:3 to C20:4). Minor allele homozygotes (7.6 % of the population) of the rs174548 variant convert C20:3 to C20:4 poly-unsaturated fatty acids (PUFAs) about 50 % slower than homozygous carriers of the major allele (52.6 % of the population). The FADS locus has been implicated in multiple GWAS with different cancers, Crohn’s disease and cardiovascular disease traits. This example shows how genetic variance in metabolic traits can be linked to complex disorders to provide a functional understanding of the underlying disease mechanism. Figure from Suhre et al. 2016

**Fig. 3:**
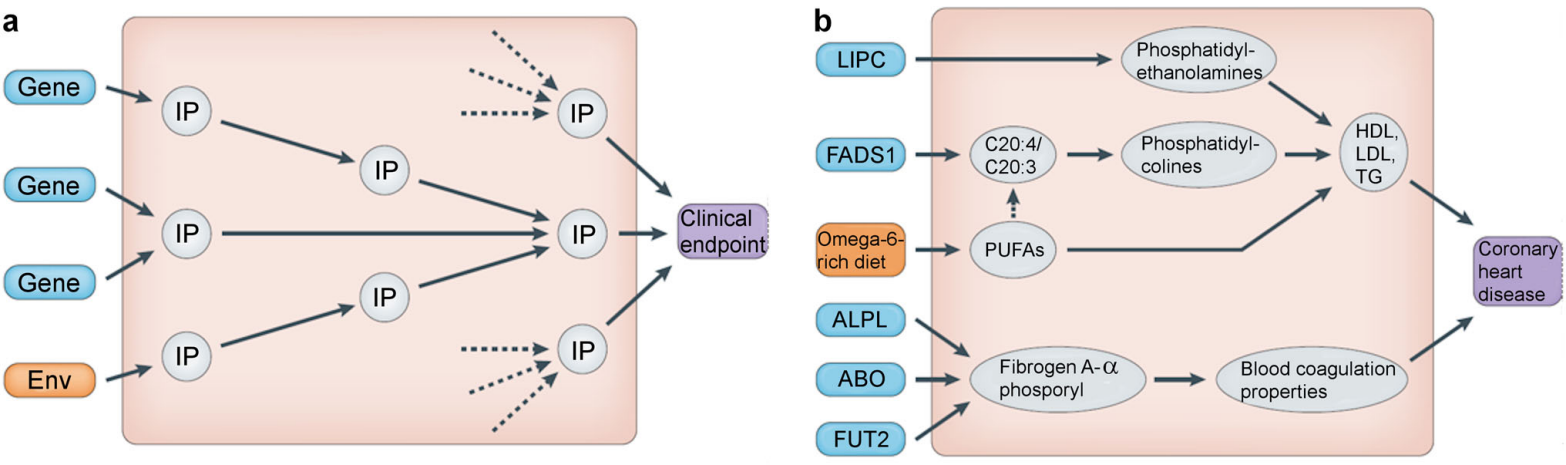
The metabolic trait is an intermediate phenotype that links the genome, lifestyle and environmental factors to the clinical endpoint. The general concept (**a**) and an example using information from actual genome-wide association studies with metabolic traits (**b**). The association of a genetic variant is strongest with its closest intermediate phenotype [IP; for example, the association of fatty acid desaturase 1 (*FADS1*) with its product–substrate pair], while the association with the clinical end point may be hard to detect at a level of genome-wide significance in a GWAS (P = 0.021 for FADS1 with coronary heart disease). The ensemble of all genetic associations with metabolic traits defines our metabolic individuality and thereby our predisposition to disease. Deep metabolic phenotyping of large precision medicine initiatives allows to identify key factors for the development of complex disorders, which can then serve as biomarkers and targets for clinical intervention. Figure reproduced from Suhre and Gieger 2012

These studies have proven Garrod’s conjecture in many instances, showing how genetic predisposition interacts through intermediate metabolic phenotypes with environmental factors and lifestyle choices in the pathogenesis of complex disorders. Figure 2 gives an example of a genetically influenced metabotype (GIM). To date, over 150 such GIMs have been discovered (Kastenmüller et al. 2015) and are now being used to dissect the genetic and environmental factors that trigger complex disorders (Fig. 3). We anticipate that deep metabolic phenotyping of the precision medicine initiative cohort program (and other precision medicine initiatives) will confidently identify fundamental factors in the development of major diseases, lead to the discovery of new biomarkers, and reveal novel targets for clinical intervention. A comprehensive blueprint of human metabolic pathways and linkage to genes and their expression, such as the global human metabolic network (Thiele et al. 2013) that had been created by the systems biology community, would inform strategies for modifying dysregulated metabolites in a rational and targeted manner, potentially using drugs that are pre-existing and safe—as suggested by recent genome-wide association study (GWAS) findings (Sanseau et al. 2012).

In summary, the metabolome provides a read out for functions of genetic variants associated with human disease and great mechanistic insights about pathways implicated in disease, disease heterogeneity, disease progression and variation in response to treatment.

### Pharmacometabolomics: a detailed biochemical roadmap for defining disease heterogeneity and drug response variation

With significant funding from the National Institute of General Medical Sciences (NIGMS), the Pharmacometabolomics and Pharmacogenomics Research Networks (PGRN and PMRN) have interacted closely over the past 8 years and pioneered how genetic and metabolic data alone, or in combination can inform about treatment outcomes and mechanisms that underlie variation in response to treatments. Over ten classes of therapies were studied in patients to illustrate the concept and its generalizability in human studies for precision medicine approach. This along with few other studies established key concepts and foundations for this new field Pharmacometabolomics (also known as Pharmacometabonomics) Table 1. A historic study in animals by Imperial researchers and their pharmaceutical consortium revealed how metabolomics data at baseline can inform about drug metabolism and toxicity (Clayton et al. 2006).

**Table 1:** Clinically-relevant and notable applications of pharmacometabolomics

Applications	Citations
Pharmacometabonomics signature predictive of drug metabolism and development of side effects to acetaminophen—role for gut microbiome	Clayton et al. 2006, 2009; Winnike et al. 2010
Metabolomics lipidomics mapping of atypical antipsychotics and baseline signature of response to three antipsychotics	Kaddurah-Daouk et al. 2007
Pharmacometabolomics and lipidomics reveals large impact of statin on lipid metabolism; lipid profile at baseline informs about treatment response that goes beyond HMGCoA reductase inhibition	Kaddurah-Daouk et al. 2010
Pretreatment metabotype as a predictor of response to antidepressant sertraline and response to placebo in depressed outpatients	Kaddurah-Daouk et al. 2011a
Gut microbiome contributes to response to simvastatin	Kaddurah-Daouk et al. 2011b
Pharmacometabolomics-informed pharmacogenomics	Ji et al. 2011
Pharmacometabolomics for cancer chemotherapies	Backshall et al., 2011; Stebbing et al. 2012; Miolo et al. 2016
Pharmacometabolomics of statin response reveals novel mechanistic insights and highlights metabolic signature for poor response	Trupp et al. 2012
Merging pharmacometabolomics with pharmacogenomics using ‘1000 Genomes’ single-nucleotide polymorphism imputation to define drug response variation to SSRI antidepressants	Abo et al. 2012
Pharmacometabolomic signatures of response to antidepressant sertraline and to placebo; insights on biochemical basis for response to placebo; biochemical insights for delayed response to SSRIs	Kaddurah-Daouk et al. 2013
Pharmacometabolomics of statin response review	Krauss et al. 2013
Pharmacometabolomics of antiplatelet therapies review	Lewis et al. 2013
Pharmacometabolomics reveals biochemical insights about ethnic differences in response to beta blocker atenolol	Wikoff et al. 2013
Pharmacometabolomics pharmacogenomics approach highlights purine pathway enzymes and genes implicated in mechanism of variation of response to aspirin	Yerges-Armstrong et al. 2013
Reviews of published work in pharmacometabolomics/pharmacometabonomics; additional references within	Wilson 2009, Everett et al. 2013; Everett 2015
Review on pharmacometabolomics a systems pharmacology approach for precision medicine	Kaddurah-Daouk et al. 2014
Pharmacometabolomics reveals a diabetes mellitus-linked amino acid signature associated with β-blocker-induced impaired fasting glucose levels	Cooper-Dehoff et al. 2014
Pharmacometabolomics reveals a role for serotonin in mechanism of varied response to aspirin treatment	Ellero-Simatos et al. 2014
Targeted lipidomics profile of aspirin’s effect on oxylipid metabolism, new mechanistic insights about response to aspirin and off target effects	Ellero-Simatos et al. 2015
Review on pharmacometabolomics enabling tools for precision medicine	Kaddurah-Daouk and Weinshilboum 2015
Pharmacometabolomic assessments of antihypertensives atenolol and hydrochlorothiazide pathways implicated in response; common and unique mechanisms	Rotroff et al. 2015
Pharmacometabolomics-informed pharmacogenomics about response to SSRI sertraline; metabolic signatures helped identify genes and SNPs implicated in response variation and disease sub classification	Gupta et al. 2016
Pharmacometabolomic assessment of metformin PK profile; pharmacometabolic signature informing about PK profile of drug	Rotroff et al. 2016
Insights from genomics and metabolomics integration on response to antihypertensives; a systems pharmacology approach for precision medicine	Shahin et al. 2016

Pharmacometabolomics evolved as a field that determines the so-called “metabotype” or metabolic state of an individual as affected by environmental, genetic, and enteric microbiome influences (Fig. 1) to study drug responses and to understand treatment outcomes. Metabolic profiles at baseline prior to treatment were shown to inform about disease heterogeneity and treatment outcomes. Also metabolic profiles provide tools for mapping global effects of drugs on metabolism, and for identifying pathways and networks implicated in the mechanisms of a drug’s action and the basis for variation in drug responses.

Examples of totally novel insights about mechanisms of variation of response to drugs used for treatment of neuropsychiatric and cardiovascular diseases were highlighted in recent reviews (Kaddurah-Daouk and Weinshilboum 2014, 2015). Metabolic profiles were shown to provide insights about variation of response to antipsychotics, statins, antidepressants, antihypertensives, antiplatelet therapies, and development of side effects to treatment. A first validation study in humans mapped effects of three antipsychotics in patients with schizophrenia, compared their effects on metabolism and defined signature at baseline implicated in treatment outcomes (Kaddurah-Daouk et al. 2007). The mapping of depressed patient’s metabotypes and the trajectory of biochemical changes induced by SSRI antidepressants (serotonin reuptake inhibitors) have since begun to explain the biochemical basis for delayed response, responses to placebo, and resistance to treatment in major depression. Ethnic basis for variation in response to antihypertensives (beta blockers and thiazide) and gender differences in response to antiplatelet therapies (aspirin) provided first examples of how metabolomics data combined with genetic data could start to define factors that contribute to variation in response to treatment. An important role for the gut microbiome was highlighted by the variation of response to statins (Kaddurah-Daouk et al. 2011b) and for acetaminophen (Clayton et al. 2006, 2009; Winnike et al. 2010). These studies as well as several others applied to the study of cancer chemotherapies (Backshall et al. 2011; Miolo et al. 2016; Stebbing et al. 2012) and others under pharmacometabonomics (for review see Everett et al. 2013; Everett 2015; Huang et al. 2015) all provide support for importance of inclusion of metabolomics data in precision medicine initiatives (Table 1). Metabolomics data captures influences on human health that go beyond genetic makeup.

**Fig. 4:**
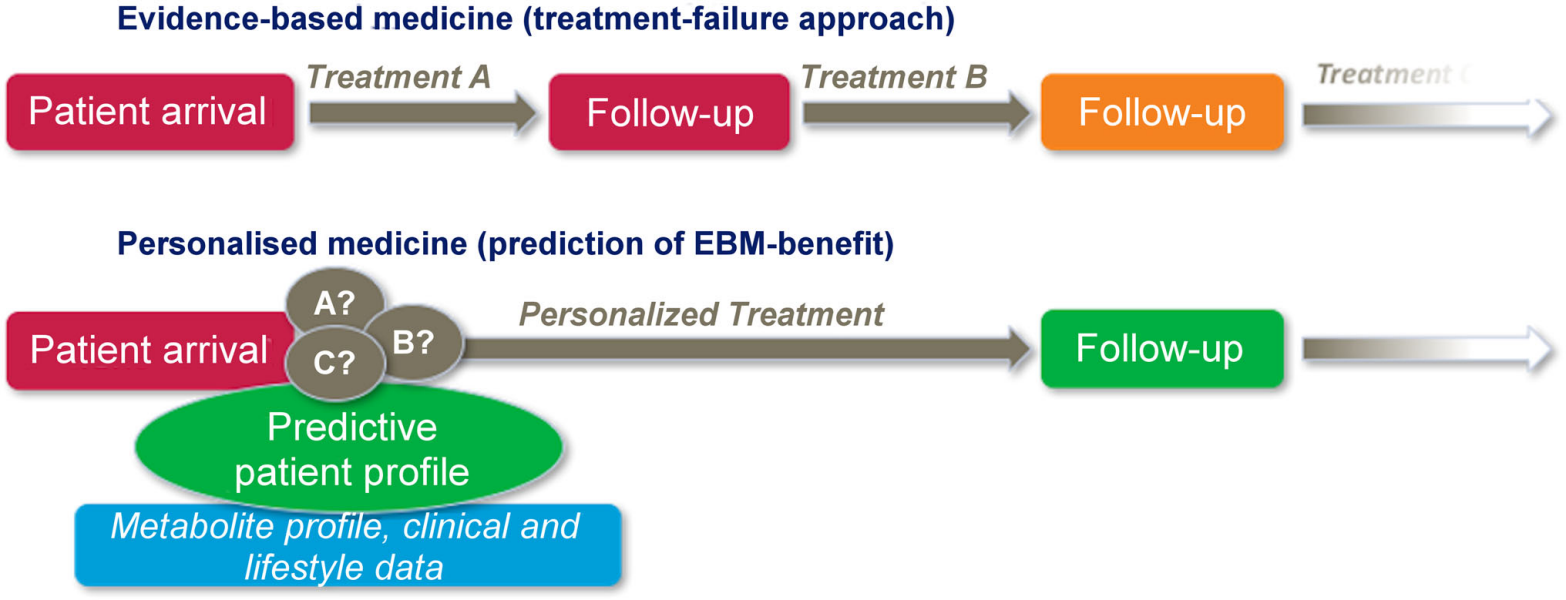
Precision medicine approach using metabolomics as compared to treatment-failure evidence-based medicine approach in clinical practice. ‘Personalized profile’ based on metabolomics as well as other clinical and lifestyle data will be used to predict the patients’ responses to specific treatments and thus help select the best treatment regimens

Metabolomics also has a great potential to be introduced directly into the clinic, as a key profiling and phenotyping platform used to predict patients’ responses to different treatments. This can lead to development of decision support tools for patients and clinicians for the purpose of selecting or recommending optimal treatment regimens (to be used in broad sense of the word, thus including also lifestyle changes). The expectation is that, by using the ‘personalized profiles,’ we will be able to circumvent the commonly applied treatment-failure approach and, thus, ultimately contribute to better patient outcomes (Fig. 4).

### The gut microflora influence human metabolism and a metabolic profile informs about gut microbiome activity

The human gut microbiome is known to possess metabolic activity considered comparable to that of the liver. The composition of the gut microbiome has wide ranging local and systemic effects. As such, it has been implicated in local disorders, such as inflammatory bowel disorders and an array of systemic conditions, including, but not limited to, disorders of the nervous, cardiovascular, and immune systems (Table 2). Microbiome-associated metabolomics has been employed to investigate and characterize a range of clinically relevant features. In this regard, it is generally considered useful to characterize both the (1) stool/gut metabolome and microbiome, in combination with (2) a readout of the host’s systemic metabolome (e.g., biofluids such as blood, urine and saliva). Clinically-relevant and notable applications of metabolomics to gut microbiome activity are noted below in Table 2.

**Table 2:** Clinically-relevant and notable applications of gut microbiome-associated metabolomics

Applications	Citations
Generating new hypotheses related to health and patterns of disease	Nicholson et al. 2005
Assessing the effect of dietary inputs on the microbiome/metabolome (protein, CHO, fat, polyphenols, etc.)	Purnbaugh and Gordon 2008; Moco et al. 2012
Identifying individual patterns of drug susceptibility, based on gut-associated metabolite patterns (e.g., urine *p*-cresol)	Clayton et al. 2009
Correlating the fecal metabolome with blood and urine metabolome to understand better peripheral markers of gut microbial metabolism	Jansson et al. 2009
Assessing the role of the microbiome in metabolizing dietary constituents to metabolically active molecules with clinical benefit (e.g., lignans and enterolactone-endocrine effects)	Peterson et al. 2010
Assessing the impact of drugs on the microbiome/metabolome (e.g., antibiotics)	Hviid et al. 2011
A role for the gut microbiome in mechanism of variation of response to statins	Kaddurah-Daouk et al. 2011b
Assessing the impact of the gut microbiome on the metabolism of prescription drugs (e.g., β-glucuronidases; effects on antibiotics, antivirals, anti-inflammatories, and anticonvulsants)	Cacciatore and Loda 2015
Assessing the effect of dietary inputs on the microbiome/metabolome relative to disease endpoints (e.g., choline, trimethylamine oxide, and CVD)	Mente et al. 2015
Correlating gut microbial genotype with small molecule molecular phenotype	Xie and Jia 2015
Correlating the gut microbial metabolome with the exposome	Patel and Manrai 2015
Understanding the patterns of microbiome-derived small molecules that enter systemic circulation	Yano et al. 2015
Developing novel small molecule biomarkers for clinical prediction	Chumpitazi et al. 2015

Thus, the metabolomics task group recommends at least one urine and one fecal sample to be collected from a subset of individuals enrolled in the PMI Cohort and other similar initiatives to evaluate gut microbiome metabolic activity. These samples would ideally be acquired following an overnight fast. Further samples, collected from a subset of the population at selected serial time points would be extremely useful for monitoring responses to environmental perturbations, e.g. how the gut microbiome is affected by cognate drug treatments.

### Nutrition-associated metabolic phenotyping

Metabolomics in a nutritional context has already been employed to investigate and characterize relevant features of the metabolic phenotype. Furthermore, diet remains a key environmental factor that needs to be characterized in order to understand its relationship to disease, and to develop a clear and confident public health message in relation to disease prevention. Disease nutrition relationships through metabolomics is not only useful for increasing our understanding of pathology, but also for understanding how the knowledge of metabolism can be employed as a key factor in disease prevention, as well as to optimize human function and performance.

Assessing dietary input accurately is difficult-most studies rely on surrogate methods based on questionnaires, which are notoriously unreliable (Dhurandhar et al. 2015). A metabolic profile of blood and urine contains biomarker information that can, in fact, provide a far more accurate estimate of dietary inputs, which can also correct misreporting on questionnaires. There is ample evidence for the importance of diet in health and disease. Obesity and adiposity have numerous effects on health including CVD and some cancers. For example, adiposity is a significant risk factor for colorectal cancer, which is higher in males than females, and is also linked to diet. Diets rich in red or processed meats increase the risk of colorectal cancer in particular (http://www.cancer.gov/about-cancer/causes-prevention/risk/obesity/obesity-fact-sheet#q6).

Therefore, nutritional metabolomics investigations fall into three major experimental categories: (A) applications to identify dietary biomarkers (B) applications to study metabolic responses to dietary interventions and (C) applications to study diet-related diseases. All of these applications can deliver further information on the impact of one of the key modifiable environmental factors (diet). Some notable applications of this approach are enumerated in Table 3.

**Table 3:** Notable applications of metabolomics in nutrition and nutrition-associated metabolic phenotyping

Applications	Citations
Evaluate the impact of nutritional status of individuals on the metabolism of drugs	Walter-Sack and Klotz 1996
Assess off-target effects of prescription drugs and the manner in which nutritional status impacts such effects	Genser 2008
Untargeted metabolome-wide association (MWA) studies for the discovery of novel biomarkers of dietary intake for disease-monitoring and accurate dietary assessment	Bictash et al. 2010
Assess the nutritional metabotype and how it correlates with metabotypes and genotypes of the microbiome in healthy and diseased participants	Bictash et al. 2010
Characterize status of phase II conjugation agents (GSH, etc.), associated with drug ingestion and adverse drug events	Johnson et al. 2012
Analyze how nutrition influences metabolism and homeodynamic control and how this regulation is disturbed in the early phase of diet-related diseases	Erazo et al. 2013
Assess essential and conditionally essential micronutrient inputs, and the spreading effect of single or multiple deficiencies (or excesses) across molecular networks	Schmidt and Goodwin 2013
Assess the connection between dietary patterns and chronic disease such as diabetes; use of dietary sensitive metabolites to explore the links between diet and disease	Zheng et al. 2015
Assess the connection between dietary patterns and high morbidity and high mortality diseases such as cancer and cardiovascular disease	Odriozola and Corrales 2015
Develop metabolomic biomarker panels associated with disease to assess disease-relevant metabotype	Gibbons et al. 2015
Analysis of food derived metabolites and their kinetics over time	Kim et al. 2016
Assess and translate metabolic changes in urine following a dietary intervention into an organ-specific, biologically meaningful interpretation and organ-specific interpretation (plasma: similar challenges present themselves)	Schmedes et al. 2016

## Metabolic profiling technologies

The revolution in the study of metabolites in the last 15 years and the development of the field of metabolomics has resulted from innovative advances in scientific instrumentation and advances in computational resources available. The continued development of chromatography coupled to mass spectrometry (MS) and nuclear magnetic resonance (NMR) spectroscopy have advanced our capabilities from monitoring only a small number of metabolites in a traditional hypothesis-testing study, to being able to simultaneously quantify hundreds to thousands of metabolites in a biological sample with an analysis time of less than 20 min (Cajka and Fiehn 2014; Fiehn 2016). This enormous new analytical capability has led to the generation of novel and completely unanticipated hypotheses (Dunn et al. 2011)—effectively shining light in places where nobody previously though to look. We define this non-targeted approach as metabolic phenotyping (“metabotyping”).

Importantly, metabotyping can provide important data not only on the metabolites present in a complex biological mixture, but also reveal molecular interactions that contribute to metabo-regulatory processes in cells and tissues. Only through the application of such holistic approaches, as a first step, can the complete biological interactome be defined in relation to human phenotypes. Once specific metabolic markers are identified, further studies applying targeted assays can be performed in attempt to validate findings and test novel hypotheses that emerge. Recent translational studies illustrate how metabotyping can lead to new and fundamental biomedical discoveries (Gooding et al. 2015; Tannahill 2013).

The current capabilities for holistic metabolic profiling apply liquid chromatography and gas chromatography coupled to mass spectrometry (GC–MS and LC–MS), as well as NMR spectroscopy. The stability and reproducibility of these platforms is hugely important in large-scale cohort studies and recent research has shown the capability now exists for robust and high quality data generation (Draisma et al. 2015; Dunn et al. 2015). This has allowed us to move from small-scale studies to large-scale investigations that may include thousands of samples. Indeed, a number of ‘Phenome Centers’ are being developed to provide the infrastructure and resources required to support large-scale studies (e.g., the National Phenome Centre in London; the Phenome Centre Birmingham UK; six NIH Regional Comprehensive Metabolomics Resource Cores in the USA and large consortia, such as Alzheimer Disease Metabolomics). Standardization of analyses performed at these centers will be a challenge, but recognized as essential to allow data to be comparable and integratable, as no single center currently provides the capacity for studies of the scale envisioned for the PMI Cohort. Without standardization there will be no ability to take data across sites and compare/integrate. These new forays have allowed knowledge from metabolic profiling studies to be synergistically applied to studies where only genomic and transcriptomic data were previously available.

Although this combined ‘omics strategy is being applied to enhance metabolic phenotyping in a holistic approach with relative quantification data created, the application of multiple (semi)-targeted assays for each sample provides a robust approach for absolute quantification of more limited metabolite panels (hundreds, not thousands). For example, the company Biocrates commercially supplies kits for analysis of targeted areas of metabolism. The Biocrates p180 kit measures amino acids, biogenic amines and 150 lipids and has been shown to be useful for evaluating dry blood spots (Biocrates Life Sciences 2016). The Biocrates bile acid kit can provide absolute quantification of 16 bile acids found in humans.

Each kit provides advantages in interlaboratory use and limitations in terms of the number of metabolites studied (where holistic untargeted approaches are far more comprehensive) and the type of quantitative information obtained (untargeted MS-based profiling provides relative differences in metabolite levels, targeted profiling provides absolute levels). Importantly, there is no single assay that can detect all metabolites present in a given sample, nor do we even currently know how many distinct molecules can be quantified. At present, the metabolome can be viewed as a biomedical frontier with important opportunities to inform on systemic processes that provide the underpinning for the coming revolution in precision medicine. Employing a combination of hydrophobic and hydrophilic chromatography with both positive- and negative-ion monitoring mass spectrometric detection, it is possible to detect 3000–4000 distinct molecules using as little as 1–20 µl of plasma (Chen et al. 2012). To take advantage of such recent analytical breakthroughs, the metabolomics community strongly recommends a combination of targeted and untargeted strategies to maximize information that can now be obtained from of an individual patient sample or sample cohort.

Major developments are expected in metabolic profiling during the next 3–5 years, as analytical approaches, compound identification, ion mobility, and derivatization agents are further developed. Major drives in metabolomics toward greater metabolome coverage, higher sensitivity and even smaller sample volumes—eventually envisioned to culminate in capabilities for single cell metabolomics. Bottlenecks are now evident in reconciling data obtained from different laboratories, though significant efforts to eliminate these are underway globally, including enhancements in data standardization, quality assurance (Dunn et al. 2012; Godzien et al. 2015) and high-confidence metabolite identification (Dunn et al. 2013); indeed the international Metabolomics Society has three separate task groups focused on driving community use and cooperation in overcoming these bottlenecks (Metabolomics Society 2014).

The type of sample being studied is always a key component in any experimental design and the choice of sample is obviously dependent on the biological question. In large-scale cohort studies, biofluids (e.g., serum, plasma, urine, saliva, stool) are most commonly employed because of their relative ease of collection, preparation and storage. No single biofluid is appropriate for all studies and the biological question defines the biofluid—oral cavity diseases match to using saliva, gut microbiome and kidney diseases match to urine whereas cardiovascular diseases and drug PK studies match to serum or plasma. K-EDTA-plasma is recommended for blood collection. Importantly the metabolome analyzed has to be representative of the metabolome collected; as metabolism operates on timescales of seconds and minutes in enzyme-containing samples and as metabolites can be chemically degraded at high temperatures or extreme pH values, it is essential that samples are collected and processed quickly to minimize any changes in metabolite composition, both qualitatively and quantitatively (Barton et al. 2008; Dunn et al. 2008). Traditionally, blood or urine samples have been collected in clinic and processed by trained staff for storage. However, this is time-consuming and costly for patients and researchers in cohort studies where travel costs, staff costs, and laboratory costs are required. One alternative is for participants in large precision medicine initiatives is to collect samples in their homes and send them frozen via express mail to a central biobank facility, thus eliminating the need for participants to travel and minimizing the efforts/costs of a trained staff. The collection of dried blood or dried urine spots on absorptive materials, followed by ambient temperature delivery by post to storage facilities, offers the potential for low-cost and larger-scale global metabotyping efforts [see cityassays.org.uk 2014 for an example for targeted analysis of vitamin D]. This emerging approach is currently being explored and may be applicable for studies during the next few years (Koulman et al. 2014; Moco et al. 2012). Notably, analysis of dried blood spots is already the standard for newborn screening of inborn errors in metabolism (Jones and Bennett 2002) and applied in many drug metabolism studies (Garcia Boy et al. 2008). The analysis of dried blood and urine spots has had limited application to holistic metabolic profiling studies as yet, but validation and adoption of this approach is anticipated.

### Stable isotope tracing in mechanism-based human health

Although tracer methodologies provide more interpretable information and power than profiling (Fan et al. 2012), this is impractical in a large study of this kind. However, such studies for a limited subset (for example, cancer) using tissue biopsies including liquid biopsies (leukocytes) can be envisaged, in which the sample is incubated with an appropriate tracer, and the metabolism of the tissue or cells is analyzed. Small amounts of tissue can now be evaluated in this manner, owing to the technological advances in mass spectrometry in particular. These approaches become especially valuable in longitudinal studies, for example pre- and post-therapy.

In summary, we have taken large steps over the last 15 years to allow the holistic study of metabolites in humans to be performed. We are now at a stage, where robust, reproducible large-scale studies of the role of metabolites as a determinant of human subject variation are becoming practical as goals of the PMI Cohort and other large initiatives. Through the combination of multiple methodologies and platforms, we can currently provide broad coverage of the metabolome and anticipate the future establishment of standardized approaches to be applied at to-be-established high-throughput metabolite profiling facilities worldwide. New developments in technology are expected to allow for an ever-broadening scope of metabolite coverage, dramatic enhancements in sample throughput, establishment of normal human ranges for diverse blood-borne metabolites and more facile ways to move samples from the home to the clinic.

### Computational medicine

Metabolic phenotyping has the potential to generate high-volumes of complex spectral data. The process of translating this data into actionable medical information, be it diagnostic, mechanistic, or patient stratification, requires significant computational power and expertise. There are currently several computational frameworks in general use in the metabolomics community; however, none of these methods are particularly focussed on the needs of precision medicine (Xia et al. 2013). As a first step, large-scale metabolic modeling permits to integrate and simulate multiple ‘omics’ data types with metabolic networks (Aurich and Thiele 2016), and it also enables to integrate dietary and genetic information in addition to metabolomic data (Heinken and Thiele 2015). The underlying metabolic models are based on human biochemistry and as such provide mechanistic links between genes, proteins, and metabolites (Aurich and Thiele 2016). There are now studies becoming available that demonstrate the potential of metabolic modeling for precision medicine (Yizhak et al. 2014), particularly when combined with personalized metabolomic data (Aurich et al. 2016). We anticipate that in the immediate future computational medicine will make a quantum leap in practical functionality, combining the robust requirements of classical epidemiology with the flexibility of modern “big data” machine learning algorithms. It will be necessary to map non-linear relationships between metabolomic, genomic, and other omic data, and combine the resulting profiles with bedside clinical metadata, producing the next generation of clinical expert systems.

Compressing such large amounts of high-throughput data into clinically coherent tools will be a major bottleneck in the development of omic precision medicine. Moreover, an omic based prognostic/diagnostic signature cannot be considered a viable “test” without an associated robust computational model. Indeed, the Institute of Medicine (IOM) report *Evolution of Translational Omics* (Institute of Medicine 2012) defines an omic’ test as “an assay composed of or derived from multiple molecular measurements and interpreted by a fully specified computational model to produce a clinically actionable result”. Thus, the development of rigorous statistical protocols together with task-specific computational models is as important to this field of research as managing the rapid advances in technology.

### Recommendations for precision medicine initiatives

1. Collect biofluids by applying metabolomics community agreed-upon standard operating procedures. Include members of the metabolomics community in working groups for establishment of optimal sample collection, preservation, sample processing, data acquisition, and omics’ analysis protocols.
2. Preserve samples by establishing a reliable and robust biobanking system with strict inventory control. Samples should include plasma (EDTA, or heparin) and preferably also serum or flash frozen samples.
3. Consider the additional collection of stool samples and urine samples for sub studies related to nutrition, gut microflora, and other scientifically relevant applications.
4. Establish resources and protocols for the distribution of biobanked samples to NIH-supported research programs or similarly funded programs.
5. Establish funding opportunities to support personalized medicine challenges using biobanked samples and both untargeted and targeted strategies for large-scale metabolic phenotyping studies.
6. Employ NIST Standard Reference material (SRM-1950) plasma samples for standardization and quality control of plasma metabolomics studies over time and across analytical sites.
7. Develop NIST Standard Reference material for urine and serum for standardization and quality control of urine and serum metabolomics studies. Develop other standard reference materials to provide additional options.
8. Establish rigorous statistical/epidemiological protocols and enable the development of the next generation of computational medicine tools.
